# Notes on the Newer Remedies, Their Therapeutic Applications and Modes of Administration

**Published:** 1893-07

**Authors:** 


NOTES ON THE NEWER REMEDIES, THEIR THERAPEUTIC APPLICATIONS AND MODES OF ADMINISTRATION, by David Cerna, M. D., Ph. D., Philadelphia. W. B. Saunders. Price, $1.25.

A very convenient little book for both the student and busy practitioner, who has not the time to read all the literature of the endless new remedies, and who often in the journals meets a remedy of which he knows nothing. While possibly not twenty-five per cent. of the remedies mentioned will stand the test of time, it may, nevertheless, be well to know something of them.

